# Study on the mechanism of antibacterial action of magnesium oxide nanoparticles against foodborne pathogens

**DOI:** 10.1186/s12951-016-0202-0

**Published:** 2016-06-27

**Authors:** Yiping He, Shakuntala Ingudam, Sue Reed, Andrew Gehring, Terence P. Strobaugh, Peter Irwin

**Affiliations:** Molecular Characterization of Foodborne Pathogens Research Unit, Eastern Regional Research Center, Agricultural Research Service, United States Department of Agriculture, 600 East Mermaid Lane, Wyndmoor, PA 19038 USA; ICAR Research Complex for NEH Region, Umiam, 793103 Meghalaya India

**Keywords:** MgO nanoparticles, Foodborne pathogens, Antimicrobial mechanism, H_2_O_2_, Oxidative stress

## Abstract

**Background:**

Magnesium oxide nanoparticles (MgO nanoparticles, with average size of 20 nm) have considerable potential as antimicrobial agents in food safety applications due to their structure, surface properties, and stability. The aim of this work was to investigate the antibacterial effects and mechanism of action of MgO nanoparticles against several important foodborne pathogens.

**Results:**

Resazurin (a redox sensitive dye) microplate assay was used for measuring growth inhibition of bacteria treated with MgO nanoparticles. The minimal inhibitory concentrations of MgO nanoparticles to 10^4^ colony-forming unit/ml (CFU/ml) of *Campylobacter jejuni*, *Escherichia coli* O157:H7, and *Salmonella* Enteritidis were determined to be 0.5, 1 and 1 mg/ml, respectively. To completely inactivate 10^8−9^ CFU/ml bacterial cells in 4 h, a minimal concentration of 2 mg/ml MgO nanoparticles was required for *C. jejuni* whereas *E. coli* O157:H7 and *Salmonella* Enteritidis required at least 8 mg/ml nanoparticles. Scanning electron microscopy examination revealed clear morphological changes and membrane structural damage in the cells treated with MgO nanoparticles. A quantitative real-time PCR combined with ethidium monoazide pretreatment confirmed cell membrane permeability was increased after exposure to the nanoparticles. In a cell free assay, a low level (1.1 μM) of H_2_O_2_ was detected in the nanoparticle suspensions. Consistently, MgO nanoparticles greatly induced the gene expression of KatA, a sole catalase in *C. jejuni* for breaking down H_2_O_2_ to H_2_O and O_2_.

**Conclusions:**

MgO nanoparticles have strong antibacterial activity against three important foodborne pathogens. The interaction of nanoparticles with bacterial cells causes cell membrane leakage, induces oxidative stress, and ultimately leads to cell death.

## Background

*Campylobacter jejuni*, *Escherichia coli* O157:H7, and *Salmonella* are the most common foodborne pathogens responsible for millions of cases of illnesses and hundreds of deaths each year in the United States [1]. *C. jejuni* is a spiral shaped and oxygen-sensitive microaerophile, whereas *E. coli* O157:H7 and *Salmonella* are rod shaped and facultative anaerobic bacteria [2]. The main natural reservoirs for these bacteria are intestinal tracts of birds (e.g. chicken) and other animals (e.g. cattle) [3–5]. Transmission of these pathogens from animal feces or the environment to food can occur during harvesting, processing, distribution, and preparation of food. Pathogen contamination has been frequently found in various food products including meat, fresh produce, dairy products, and ready-to-eat foods. The sporadic prevalence of microbial pathogens in food and increased incidence of antibiotic resistant strains have posed serious concerns to public health [6, 7]. Hence, there is a need to develop alternative strategies for effective control of microbial pathogens in food and the environment.

Metal oxide nanoparticles such as ZnO, MgO, CuO, CaO, Ag_2_O, and TiO_2_ are a new class of antimicrobial agents that have been increasingly studied for their antibacterial properties and potential applications in food, the environment, and healthcare [8, 9]. As nanoscale (<100 nm) inorganic materials, metal oxide nanoparticles have distinct features including broad spectrum antibacterial activity, large surface area of interaction with cells, low possibility for bacteria to develop resistance, high stability even under harsh conditions, and tunable sizes, shapes, surface properties, and chemical compositions, which lead to great potential for developing nanomaterials as effective antimicrobial agents. Among these, nanostructured MgO is particularly interesting due to its strong antibacterial activity, but high thermal stability and low cost.

The mechanism of metal oxide nanoparticle action on bacteria is complicated and not fully understood. It has been reported that the antibacterial activity of MgO nanoparticles is attributed to the production of reactive oxygen species (ROS) which induce lipid peroxidation in bacteria [10]. In contrast, non-ROS mediated bacterial toxicity was also found in MgO nanoparticles, suggesting oxidative stress might not be the primary mechanism of cell death [11]. Furthermore, the antibacterial effect not only depends upon the sizes, shapes, chemical composition, and surface properties (e.g. hydrophobicity) of the nanoparticles, but also varies with bacterial species [12, 13]. Several studies have shown that smaller particles have greater antibacterial activity due to higher reactive surface area [9]. However, aggregations of very small nanoparticles (~5 nm) could reduce the efficiency of interaction with bacteria. It also has been reported that MgO and CuO nanoparticles had substantially higher antibacterial activities on Gram-positive (G+) than Gram-negative (G−) bacteria, presumably due to the differences in cell membrane structure between these organisms. Our previous study showed that ZnO nanoparticles displayed extremely strong activity against *C. jejuni* compared to *E. coli* O157:H7 or *Salmonella* likely due to the different tolerances of these organisms to oxidative stress induced by nanoparticles [13].

Cytotoxicity is a major concern in the development of antimicrobial agents. MgO has been used as a mineral supplement for magnesium, an essential nutrient for the human body. As a medicine, MgO is used for the relief of cardiovascular disease and stomach problems. At low concentrations (0.3 mg/ml), MgO nanoparticles were reported to not be toxic to human cells [14]. However, toxic effects are greatly dependent on the physical and chemical properties of nanoparticles as well as the types of cells tested [15, 16]. Hence, extensive evaluation of nanoparticles on different biological systems is needed to determine the toxicity of nanoparticles. Understanding of the mechanism of nanoparticle action on bacteria could provide useful guidelines for rational design and assembly of effective antibacterial derivatives. Advanced strategies, such as packaging multiple antimicrobial agents into the same nanoparticle, coating nanoparticles with biodegradable materials, and engineering target-specific nanoparticles for delivery to infection site, have emerged to improve antimicrobial activities and reduce undesirable side effects of nanoparticles.

The aim of this research was to study the antimicrobial activity and mechanism of action of MgO nanoparticles on three major foodborne pathogens. Through scanning electron microscopy (SEM) examination of cell morphology and membrane structure, ethidium monoazide combined with quantitative real-time PCR (EMA-qPCR) measurement of membrane permeability, transcriptional analysis of oxidative stress defense genes, and quantification of H_2_O_2_ produced in nanoparticle suspensions, we suggested the most conceivable mechanism of action of MgO nanoparticles on bacteria.

## Methods

### MgO nanoparticles

MgO nanoparticles with an average size of 20 nm were purchased from Nanostructured & Amorphous Materials, Inc. (Houston, TX, USA). ZnO nanoparticles (average size of 30 nm) were from Inframat Advanced Materials LLC (Manchester, CT, USA). A stock suspension (8 mg/ml) was freshly prepared by resuspending 80 mg of the nanoparticles into 10 ml ddH_2_O for H_2_O_2_ production assay or Mueller Hinton broth (MH broth; Becton–Dickinson Co., Sparks, MD, USA) for cell culture experiments. All of the nanoparticle suspensions were homogenized by vigorous vortexing prior to use in the following experiments.

### Bacterial culture conditions

*Campylobacter jejuni* 81–176 was statically grown at 42 °C in MH broth in a microaerophilic workstation (Don Whitley Scientific, Ltd., Shipley, UK) maintaining an atmosphere of 5 % O_2_, 10 % CO_2_, 85 % N_2_, and 82 % relative humidity. *Salmonella enterica* serovar Enteritidis ATCC 13076 and *E. coli* O157:H7 EDL 933 were aerobically grown at 37 °C in MH broth with shaking at 190 revolution per minute (rpm) (Innova 42, New Brunswick, Enfield, CT, USA).

### Minimum inhibitory concentration of MgO nanoparticles

The viability of bacterial cells when exposed to varying concentrations of MgO nanoparticles was analyzed in a 96-well plate using the Resazurin Cell Viability Assay Kit (Bio Trend Chemicals LLC, Destin, FL, USA). Resazurin indicates cell viability by changing from a blue/non-fluorescent state to a pink/highly fluorescent state upon chemical reduction resulting from aerobic respiration due to cell growth. Overnight cultures of *C. jejuni*, *E. coli* O157:H7, and *S*. Enteritidis were diluted to approx. 10^4^ CFU/ml. MgO nanoparticles were diluted 1:2 in MH broth from a starting concentration of 8 mg/ml in successive columns of a microtiter plate to an ending concentration of 0.03 mg/ml. To each well containing 100 μl of MgO nanoparticle suspension and 100 μl of the diluted bacteria, 20 μl of Resazurin dye was added and mixed thoroughly. Each microorganism was tested in a different plate with eight replicates of each concentration of MgO nanoparticles. Three controls without nanoparticles (100 μl each of MH broth without cells, and live and heat-killed cells at 10^4^ CFU/ml) were also included in each plate. The plates were then incubated aerobically at 37 °C for *E. coli* and *Salmonella* or under microaerophilic conditions overnight at 42 °C for *Campylobacter*. After incubation, the plates were subjected to fluorescence measurement at an excitation wavelength of 530 nm and emission wavelength of 590 nm using a Tecan Safire2™ microplate reader (Männedorf, Switzerland) as well as visual inspection for color change. The lowest concentration of nanoparticle suspension that inhibited cell growth (dye did not convert to red) was defined as the minimum inhibitory concentration (MIC).

### Antimicrobial effects of MgO nanoparticles

The antimicrobial effects of MgO nanoparticles were studied by exposing 10^8−9^ CFU/ml of *C. jejuni*, *E. coli* O157:H7, and *S.* Enteritidis to 0, 0.5, 1, 2, 4 and 8 mg/ml nanoparticles. The samples were incubated aerobically at 37 °C for *E. coli* and *Salmonella* or microaerobically at 42 °C for *Campylobacter* for a total of 24 h. At specific time intervals (0, 0.5, 1, 2, 4, 6 and 8 h), 1 ml of each sample was collected to determine colony-forming units (CFU) on MH agar by the 6 × 6 drop plate method [17]. The average number of CFU/ml (log_10_) and standard deviation from 6 replicates were used to plot each data point.

### Morphological analysis by scanning electron microscope

Bacterial cultures of *E. coli* O157:H7, *S*. Enteritidis, and *C. jejuni* were treated with 0, 1 and 2 mg/ml MgO nanoparticles for 8 h. Aliquots of 1 ml samples were centrifuged for 2 min at 4000 rpm and the cell pellets were resuspended in 0.1 ml MH broth. Subsequently, 20 μl of each concentrated sample was deposited and spread onto a glass coverslip pre-washed with acetone and ethanol. After drying the slips for 15 min at 37 °C, the bacterial cells were subjected to fixation, dehydration, and critical point drying for SEM analysis as described previously [13].

### EMA-qPCR assessment of cell membrane integrity

EMA-qPCR analysis of *C. jejuni*, *E. coli* O157:H7, and *S*. Enteritidis cells treated and untreated with MgO nanoparticles was carried out as described previously [18]. Briefly, late log phase cultures of *Campylobacter* were treated with 0, 1 and 2 mg/ml of MgO nanoparticles for 8 h at 42 °C in microaerophilic conditions. *E. coli* and *Salmonella* were treated with 0, 2 and 4 mg/ml of nanoparticles for 8 h aerobically at 37 °C. After incubation, 1 ml of each sample was treated with 20 μg/ml EMA in the dark for 5 min and subsequently exposed to a 600 W halogen light for 1 min on ice. Cells were immediately centrifuged at 8000 rpm for 5 min and washed with phosphate-buffered saline. Genomic DNA was extracted from the samples using the DNeasy Blood and Tissue kit (Qiagen, Valencia, CA, USA) and qPCR analyzed using a 7500 Real-Time PCR system (Applied Biosystems, Carlsbad, CA, USA). In the qPCR assay, organism-specific gene targets (*hipO*, *rfbE*, and *invA*), primers and TaqMan probes, and DNA standard curves were chosen for the detection of *C. jejuni*, *E. coli*, and *Salmonella*, respectively [18, 19]. The threshold cycle (Ct) values obtained from the EMA-qPCR assay were converted to DNA copy numbers based on the linear regression equations of the DNA standard curves. The copy number of the DNA target is equivalent to the genome copy of the bacterial cells and provides a good estimate for the number of cells.

### Gene expression analysis of nanoparticle treated and untreated cells

Total cellular RNA was extracted from 100 ml of late-log phase (13 h microaerobic incubation) *C. jejuni* culture treated with 0 and 1 mg/ml MgO nanoparticles for 30 min by using TRI-Reagent^®^ (Molecular Research Center, Inc. Cincinnati, OH, USA). DNase I treatment and reverse transcription of the RNA samples were carried out as previously described [20]. The expression of stress response genes was quantified by a real-time PCR assay using SYBR green master mix (Applied Biosystems, Foster City, CA, USA) and the primer sets described in our previous report [13]. Housekeeping genes *tsf*, *gyrA*, and *16S rRNA* were included as references for data normalization. The difference of gene expression between nanoparticle treated and untreated cells was calculated using 2^−ΔΔCt^ formula: ΔΔCt = ΔCt (treated sample) −ΔCt (untreated sample), ΔCt = Ct (target gene) −Ct (*16S rRNA*), and Ct is the threshold cycle value of the amplified target or reference gene [21].

### Quantification of H_2_O_2_ production in MgO and ZnO nanoparticle suspensions

The assay of H_2_O_2_ released from nanoparticle suspensions was performed in a 96-well plate using the Red Hydrogen Peroxide Assay Kit (Enzo Life Sciences, Inc. Farmingdale, NY, USA). The assay utilizes horseradish peroxidase (HRP) to catalyze the conversion of a red peroxidase substrate into resorufin (a highly colored and fluorescent compound) during the reduction of H_2_O_2_ to O_2_ and H_2_O. Scanning of resorufin absorbance was used for the detection of H_2_O_2_ production. Briefly, a 5 ml reagent mix was prepared by adding 50 μl of red peroxidase substrate (100×) and 200 μl of HRP (20 units/ml) into 4.75 ml assay buffer. To each well of a microtiter plate, 100 μl each of the freshly prepared reagent mixture and nanoparticle suspensions (0.5, 1, 2 and 4 mg/ml) were added. After 1-h incubation at room temperature, the plate was centrifuged at 4000 rpm for 5 min to pellet the nanoparticles. Subsequently, 100 μl supernatant from each well was transferred into an ultraviolet (UV) transparent plate and scanned for absorbance between 475 and 600 nm wavelengths using a Tecan Safire2™ microplate reader. To estimate the H_2_O_2_ concentrations in the nanoparticle suspensions, a standard curve was prepared using a set of 1:3 serial dilutions of H_2_O_2_ in distilled water (10, 3.33, 1.11, 0.37, 0.12, 0.04 and 0.01 μM) and scanned in the same assay. Distilled water was used as a blank for scanning. Each of the samples including the standards were analyzed in duplicate.

## Results

### Minimum inhibitory concentration of MgO nanoparticles

**Fig. 1:**
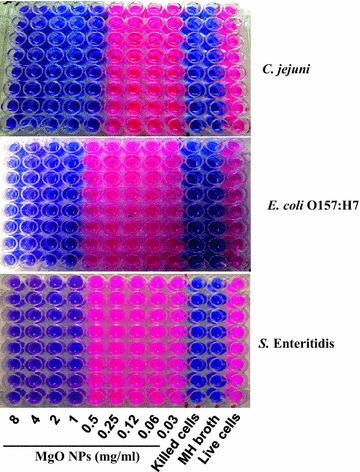
MIC of MgO nanoparticless for *C. jejuni*, *E. coli* O157:H7, and *S.* Enteritidis. Freshly grown and diluted bacterial cultures (approx. 10^4^ CFU/ml) were treated with various concentrations of MgO nanoparticles. The visible color change from *nonfluorescent blue* (resazurin) to *fluorescent red* (resorufin) shows reductive activities of the cells, indicating no inhibition on cell growth

The viability of *C. jejuni*, *E. coli* O157:H7, and *S.* Enteritidis when exposed to varying concentrations of MgO nanoparticles was determined using an oxidation–reduction assay. Resazurin, a redox sensitive dye, was used to indicate cell viability. Metabolically active cells reduce the non-fluorescent blue resazurin to fluorescent red resorufin. Non-living cells do not reduce the resazurin, and thus indicate cell death. This visible change in color and fluorescence indicates the cells are viable. Freshly grown and diluted bacterial cultures in MH broth (approx. 10^4^ CFU/ml) were exposed to MgO nanoparticles at concentrations ranging from 0.03 to 8 mg/ml. *C. jejuni* cells were rendered non-viable after treatment with 0.5 mg/ml of MgO nanoparticles. *E. coli* O157:H7 and *S.* Enteritidis were inactivated with 1 mg/ml of MgO nanoparticles (Fig. 1). This indicates that *C. jejuni* is more susceptible to the antimicrobial effects of MgO nanoparticles than *E. coli* O157:H7 or *S*. Enteritidis.

### Lethal effect of MgO nanoparticles

**Fig. 2:**
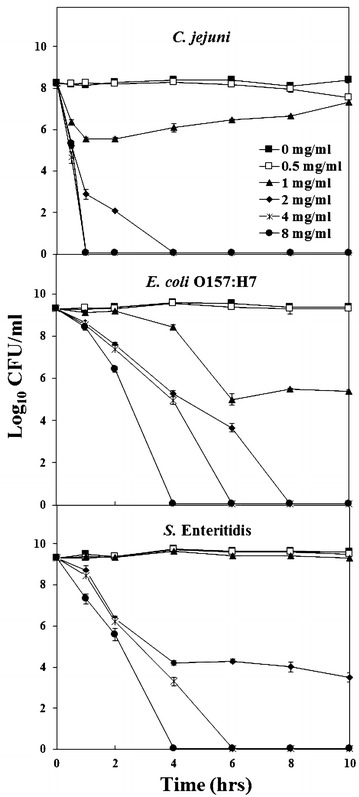
Antimicrobial effect of MgO nanoparticles against *C. jejuni*, *E. coli* O157:H7, and *S.* Enteritidis. Various concentrations of nanoparticles were applied to approx. 10^8^ CFU/ml of *C. jejuni* and 10^9^ CFU/ml of *E. coli* O157:H7 or *S.* Enteritidis. At different times after the treatment, viable cell counts were measured by culturing bacterial colonies on MH agar plates. Each of the CFU/ml value represents the mean of six replicates

The observed antimicrobial effect was further investigated by exposing 10^8^ CFU/ml *C. jejuni* and 10^9^ CFU/ml *E. coli* O157:H7 and *S.* Enteritidis to 0, 0.5, 1, 2, 4 and 8 mg/ml MgO nanoparticles over a set time trial (Fig. 2). Live cells were measured by the colony forming units on MH agar. At a concentration of 2 mg/ml MgO nanoparticles, *C. jejuni* was reduced 6 orders of magnitude after 2 h and completely killed after 4 h. At 4 mg/ml, *C. jejuni* was completely killed within 1 h. On the contrary, 8 mg/ml MgO nanoparticles were required to kill all *E. coli* O157:H7 and *S. Enteritidis* cells in 4 h and 4 mg/ml in 6 h. In addition, *E. coli* O157:H7 could also be killed by 2 mg/ml in 8 h, whereas *S*. Enteritidis was only reduced 5 logs after the same exposure. This demonstrates again that MgO nanoparticles are effective at killing *C. jejuni* at low concentrations in short periods of time. They are also advantageous at killing *E. coli* O157:H7 and *S*. Enteritidis within 4 h.

### MgO nanoparticles alter bacterial cell morphology and membrane structure

**Fig. 3:**
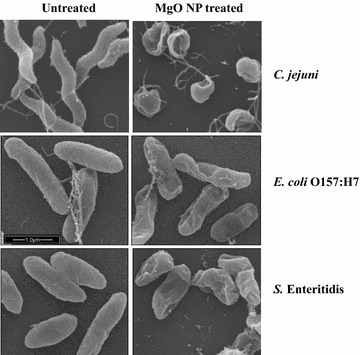
Scanning electron micrographs of *C. jejuni*, *E. coli* O157:H7, and *S.* Enteritidis. SEM images were taken from the bacterial cells of *C. jejuni*, *E. coli* O157:H7, and *S.* Enteritidis treated (*right panel*) with 2 mg/ml MgO nanoparticles for 8 h. The control cells (*left panel*) were incubated under the same conditions without adding nanoparticles

To explore antimicrobial mechanism of the nanoparticles, scanning electron microscopy was used to examine the morphological and membrane structure changes of *C. jejuni*, *E. coli* O157:H7, and *S.* Enteritidis induced by MgO nanoparticles. Bacterial cells in late-log growth were treated with sub-lethal doses of MgO nanoparticles (1 and 2 mg/ml) for 4 h and collected for SEM study. Both treated and untreated cells were incubated under the same conditions and analyzed by SEM in parallel in order to observe the differences between the control and cells exposed to nanoparticles. SEM images in Fig. 3 show all of the untreated cells have intact and smooth surfaces. As expected, *C. jejuni* cells are spiral-shaped, whereas *E. coli* O157:H7 and *S.* Enteritidis are rod-shaped. After incubation with a sub-lethal concentration of nanoparticles, *C. jejuni* cells underwent significant morphological changes from spiral to coccoid form, but *E. coli* O157:H7 and *S.* Enteritidis remained rod shaped. Noticeably, all of the treated cells displayed some deep craters on their membrane surface, indicating a degree of membrane structure damage. These cells appear to be shorter and more compact, suggesting there could be some leakage of the cellular contents caused by the treatment. No cell lysis was noticed after the treatment of sub-lethal concentrations of nanoparticles.

### MgO nanoparticles increase *C. jejuni* membrane permeability

**Fig. 4:**
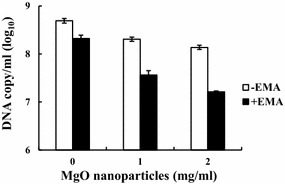
EMA-qPCR analysis of *C. jejuni* membrane permeability. *C. jejuni* cells exposed to 0, 1 or 2 mg/ml of nanoparticles were measured for the inhibition of DNA amplification caused by the penetrated EMA. The inhibition of DNA amplification was monitored by qPCR of the *hipO* gene. In the presence of EMA, reduced DNA amplification indicates increased membrane permeability in the cells

To further investigate the effect of MgO nanoparticles on cell membrane integrity and permeability, ethidium monoazide-qPCR assay was employed. Ethidium monoazide (EMA) is a DNA-binding intercalator that enters cells through compromised membranes. After photo cross-linking, the intercalation between EMA and cellular DNA becomes irreversible due to the formation of intermolecular covalent bonds. The EMA-bound DNA cannot be amplified by PCR, thus indicating membrane damage. The membrane permeability of *C. jejuni* after exposure to 1 and 2 mg/ml MgO nanoparticles for 4 h was assessed by EMA-qPCR assay. The results in Fig. 4 show that cells exposed to MgO nanoparticles had a nearly 1-log reduction in DNA amplification, indicating EMA penetration via damaged membranes. Similar experiments were performed on *E. coli* O157:H7 and *S.* Enteritidis cells after exposure to 2 and 4 mg/ml MgO nanoparticles. The effects of membrane leakage by MgO nanoparticles were less noticeable compared to *C. jejuni* (data not shown). Together, these results indicate that MgO nanoparticles increase cell membrane permeability and that *C. jejuni* is more susceptible to the membrane damage than *E. coli* O157:H7 and *S. Enteritidis.*

### MgO nanoparticles induce the expression of oxidative stress response genes in *C. jejuni*

**Fig. 5:**
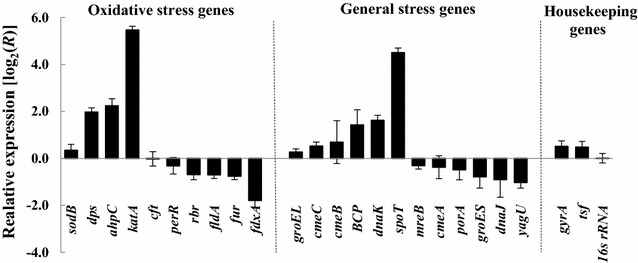
Difference of stress gene expression between MgO nanoparticle treated and untreated *C. jejuni*. Late-log phase cells treated with 0 or 1 mg/ml MgO nanoparticles for 30 min were quantified for mRNA of stress genes by reverse transcription-qPCR. Relative expression ratio (ΔΔCt) of each gene is presented in a log_2_ value

To study the molecular mechanism of MgO nanoparticle action on bacteria, expression of the genes involved in oxidative and general stress defenses was examined in *C. jejuni*. Late-log phase cells exposed to 1 mg/ml MgO nanoparticles for 30 min were collected for transcription analysis. Transcripts/mRNAs of these genes were prepared and quantified by reverse transcription-qPCR. In response to the treatment, the expression of oxidative stress response genes *katA* (encoding catalase), *ahpC* (encoding alkyl hydroperoxide reductase), and *dps* (encoding bacterioferritin) were upregulated 44-, 5- and 4-fold, respectively (Fig. 5). In addition, general stress response gene *spoT* [encoding a bifunctional (p) ppGpp synthetase/hydrolase] was also expressed 22-fold higher. As controls, transcriptions of housekeeping genes *gyrA*, *tsf*, and 16 s rRNA were not significantly affected. *katA* is the sole catalase gene present in *C. jejuni* and is essential for resistance to hydrogen peroxide [22]. *spoT* is a stringent response gene required for survival under high O_2_ [23]. Dramatic increase of the catalase gene (*katA*) and stringent response gene (*spoT*) expression in response to treatment strongly suggests MgO nanoparticles induce oxidative stress in *C. jejuni* cells. To survive, bacteria regulate their detoxification system by increasing their level of oxidative stress defense proteins as well as some of the general stress response proteins.

### Nanoparticle suspensions generate hydrogen peroxide

**Fig. 6:**
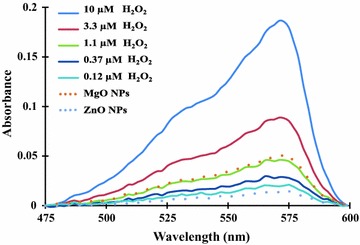
Production of H_2_O_2_ in MgO/ZnO nanoparticle suspensions. The production of H_2_O_2_ in nanoparticle suspensions was measured by spectral absorbance scanning of resorufin, a fluorescent compound formed from a peroxidase substrate by H_2_O_2_ reduction. The solid curves represent the absorbance of various known concentrations of H_2_O_2_ standards, and the dotted curves show the levels of H_2_O_2_ released from MgO/ZnO nanoparticle suspensions after baseline subtraction. The maximum excitation and emission wavelengths of resorufin fluorescence are at 570 and 585 nm, respectively

To further investigate the causes of oxidative stress in bacteria by MgO nanoparticles, we measured hydrogen peroxide (H_2_O_2_) produced in nanoparticle suspensions by using the highly sensitive Red Hydrogen Peroxide assay kit (with sensitivity as low as 10 pmol). H_2_O_2_ contains a highly reactive oxygen species and is able to penetrate into cells to cause oxidative stress. Since H_2_O_2_ is the most unstable form of reactive oxygen species, all the nanoparticle suspensions and H_2_O_2_ standard solutions were freshly prepared for the study. The levels of H_2_O_2_ produced in nanoparticle suspensions were determined by scanning the absorbance of resorufin fluorescence between 475 and 600 nm wavelengths and shown in Fig. 6. Referenced to the fluorescent absorbance of known concentrations of H_2_O_2_ standards (0.12, 0.37, 1.1, 3.3 and 10 μM), the H_2_O_2_ released in MgO nanoparticle suspension was estimated to be ca. 1.1 μM. In the same assay, a low level of H_2_O_2_ (ca. 0.12 μM) was also detected in ZnO nanoparticle suspensions, suggesting the H_2_O_2_ generated from MgO/ZnO nanoparticles contributed to antibacterial activity by inducing oxidative stress in cells.

## Discussion

In this study, we have shown that MgO nanoparticles have a strong antimicrobial activity against three major foodborne pathogens from both the microplate-based resazurin assay and viable cell count method. The use of visible color change of resazurin from blue to red as an indicator of cell growth not only made the detection simple and fast, but allowed us to avoid the turbidity problem of insoluble nanoparticles interfering with cell optical density measurements. By using this assay, we determined that the minimal inhibitory concentrations of MgO nanoparticles against 10^4^ CFU/ml of *C. jejuni* and *E. coli* O157:H7/*Salmonella* were 0.5 and 1 mg/ml, respectively. This is reasonably close to the result reported by Krishnamoorthy et al. [24] which showed the MIC of MgO nanoparticles (average size 25 μM) to 5 × 10^5^ CFU/ml of *E. coli* was 0.5 mg/ml. The slight variances of the MICs found in these studies might be due to the size and/or shape variations of the nanoparticles or the different numbers of cells used.

By viable cell count, we found at least 2 mg/ml nanoparticles (equivalent to 1.3 × 10^14^ of 20 nm size and sphere-shaped MgO nanoparticles per ml) were required to completely inactivate 10^8^ CFU/ml of *C. jejuni*, and 4 mg/ml was the minimal amount of nanoparticles needed to kill 10^9^ CFU/ml of *E. coli* O157:H7 or *Salmonella*. Apparently, higher concentrations of MgO nanoparticles were required to inactivate increased numbers of bacterial cells. Also, *C. jejuni* cells were found to be more susceptible to the nanoparticles than *E. coli* O157:H7 or *Salmonella*.

In this study, we chose *C. jejuni*, a microaerophilic bacterium, as a model organism to study the expression of stress defense genes. *C. jejuni* is extremely sensitive to oxidative stress due to the lack of SoxRS and OxyR, the most important regulatory proteins of oxidative stress defense in *E. coli* and *Salmonella*. It has been known that *C. jejuni* uses a number of enzymes including KatA, SodB, AhpC, Dps, Tpx, and Bcp to detoxify a low level of endogenous H_2_O_2_ produced during cell metabolism and as a defense to the oxidative stresses potentially encountered in a host and the environment [25]. To better understand bacterial cell responses to the nanoparticle treatment, we selected a number of genes associated with oxidative stress and general stress defenses as targets of transcriptional study. Of the important anti-oxidative stress proteins in *C. jejuni*, KatA is the only catalase and primary enzyme for decomposing H_2_O_2_ to H_2_O and O_2_; AhpC is the secondary enzyme for reducing H_2_O_2_ and is responsible for removal of low level of H_2_O_2_ from cells; and Dps protects cellular DNA from oxidative damage [26–28].

From transcriptional analysis of the stress response genes, we found the expression of *katA*, *ahpC*, and *dps* were significantly increased in response to a sub-lethal concentration of MgO nanoparticle treatment. Interestingly, the same set of stress response genes (*katA*, *ahpC*, and *dps*) were also found expressed at higher levels in the *C. jejuni* cells treated with 1 mM H_2_O_2_ [29]. H_2_O_2_ is highly reactive to cell biomolecules (e.g. DNA, proteins, and lipids) and causes oxidative stress in bacteria when present at high concentrations. Severe or continuous oxidative stress could result in failure of the cellular defense system and cell death. By using a highly sensitive cell-free assay, we detected approximately 0.12 and 1.1 μM H_2_O_2_ in ZnO and MgO nanoparticle suspensions, respectively. The concentrations of H_2_O_2_ produced in these nanoparticle suspensions seems to be relatively low, so it is possible that the production of H_2_O_2_ from nanoparticles is not the sole mechanism for the antibacterial activity of MgO/ZnO nanoparticles. SEM analysis showed considerable cell morphology change and membrane disruption in the cells treated with MgO nanoparticles. Furthermore, the EMA-qPCR results provided from this study confirmed that MgO nanoparticles increased membrane permeability in the bacteria which likely resulted in the leakage of cell content.

Our previous study showed ZnO nanoparticles had remarkable anti-*Campylobacter* activity with the MIC 8–16 fold lower than *E. coli* and *Salmonella* [13] In this study, we did not find significant differences of MgO nanoparticles in inhibiting growth or inactivating cells between *C. jejuni*, *E. coli* O157:H7, and *S.* Enteritidis. However, the effects of ZnO and MgO nanoparticles on triggering cell morphology change, membrane leakage, and oxidative stress, were found to be similar, suggesting the modes of actions of these nanoparticles on bacteria might be similar but not exactly the same.

On the basis of these findings, we propose multiple mechanisms for the action of MgO nanoparticles on bacteria: (1) MgO nanoparticles continuously generate a certain level of H_2_O_2_ while in suspension, which induces oxidative stress in cells; (2) physical interaction between nanoparticles and cell surface disrupts bacterial membrane integrity and causes membrane leakage; (3) higher concentrations of nanoparticles lead to severe membrane damage, cell content release, irreversible oxidization of biomolecules (e.g. DNA. proteins, and lipids), and ultimately cell death.

## Conclusions

This study demonstrated that MgO nanoparticles have strong antibacterial activity against three important foodborne pathogens and addressed the underlying mechanisms of MgO nanoparticles’ deleterious action on bacteria. The distinct evidence of H_2_O_2_ production in suspension of MgO nanoparticles, nanoparticle-induced expression of oxidative stress defense genes, alteration of cell morphology, and membrane leakage strongly suggests that the antibacterial mechanism of MgO nanoparticles is due to the induction of oxidative stress and disruption of membrane integrity in bacterial cells.

## Abbreviations

CFU: colony forming unit
*C. jejuni*: *Campylobacter jejuni*
*E. coli*: *Escherichia coli*
*S*. Enteritidis: *Salmonella* Enteritidis
KatA: catalase
ROS: reactive oxygen species
G+: Gram positive
G−: Gram negative
SEM: scanning electron microscopy
EMA: ethidium monoazide
qPCR: quantitative real-time PCR
MH: Mueller–Hinton
MIC: minimum inhibitory concentration
rpm: revolution per minute
ATCC: American Type Culture Collection
Ct: threshold cycle
HRP: horseradish peroxidase
UV: ultraviolet
